# Improved preculture management for *Cupriavidus necator* cultivations

**DOI:** 10.1007/s10529-023-03436-1

**Published:** 2023-10-13

**Authors:** Michelle-Sophie Gerlach, Peter Neubauer, Matthias Gimpel

**Affiliations:** Chair of Bioprocess Engineering, Institute of Biotechnology, Technische Universität Berlin, Ackerstr. 76, ACK24, 13355 Berlin, Germany

**Keywords:** *Cupriavidus necator*, NAD^+^-reducing hydrogenase, Preculture management, Bioprocess development

## Abstract

**Objectives:**

Research on hydrogenases from *Cupriavidus necator* has been ongoing for more than two decades and still today the common methods for culture inoculation are used. These methods were never adapted to the requirements of modified bacterial strains, resulting in different physiological states of the bacteria in the precultures, which in turn lead prolonged and different lag-phases.

**Results:**

In order to obtain uniform and always equally fit precultures for inoculation, we have established in this study an optimized protocol for precultures of the derivative of *C. necator* HF210 (*C. necator* HP80) which is used for homologous overexpression of the genes for the NAD^+^-reducing soluble hydrogenase (SH). We compared different media for preculture growth and determined the optimal time point for harvest. The protocol obtained in this study is based on two subsequent precultures, the first one in complex nutrient broth medium (NB) and a second one in fructose –nitrogen mineral salt medium (FN).

**Conclusion:**

Despite having two subsequent precultures our protocol reduces the preculture time to less than 30 h and provides reproducible precultures for cultivation of *C. necator* HP80.

## Introduction

*Cupriavidus necator* (formerly *Ralstonia eutropha*), aside from being a major producer of pholyhydroxyalkanoates, has become a model organism for lithoautotrophic growth with H_2_ and CO_2_ (Burgdorf et al. 2005; Lenz et al. 2002; Schwartz et al. 2009) but also its ability to produce polyhydroxybutyrate (PHB) (Budde et al. 2010; Riedel et al. 2012) and its oxygen-tolerant [NiFe] hydrogenases (Lenz et al. 2002; Poladyan et al. 2019; Schäfer et al. 2016) are in the focus of current research. Especially, the energy-converting hydrogenases are of particular interest. They are involved in H_2_ oxidation thus making them promising candidates for H_2_-driven biotransformations, e.g. as part of co-factor regeneration systems, biosensors, or as bioanodes in biofuel cells (Burgdorf et al. 2005; Jugder et al. 2016b; Lauterbach and Lenz 2013, 2019; Lenz et al. 2015; Lu et al. 2016; Schneider and Schlegel 1976). Hence, it is important to find time- and cost-saving biotechnological processes for production of high yields of these hydrogenases. So far, different approaches to obtain NAD^+^-reducing active soluble hydrogenase (SH) from *C. necator* have been used, however, experiments have only been performed at laboratory scale up to a volume of 5 L (Jugder et al. 2016b; Lauterbach and Lenz 2013; Lenz et al. 2018). For time and cost saving and for application to industrial scales it is important to establish a reproducible standard to obtain precultures that leads to fast growing main cultures, which in the end results in large amounts of SH.

For almost 50 years, not much has changed in the standard cultivation methods of *C. necator*. Thus, the standard method for precultures is inoculation with fresh transformants from agar plates into liquid fructose-nitrogen medium (FN) (Kleihues et al. 2000; Schlegel et al. 1961). Over the years, only the used medium was modified in its composition, e.g. fructose was added for heterotrophic growth (Lenz et al. 1994; Schäfer et al. 2013). From the beginning, precultures were incubated at 30 °C, but in between, 37 °C was repeatedly chosen as incubation temperature (Al-Shameri 2020; Karstens 2014; Poladyan et al. 2019; Wilkening 2021). Incubation times were also continuously changed; common precultures are either performed as overnight cultures, (Jugder et al. 2016b) or are cultivated for up to 48 h (Goris 2011; Karstens 2014; Schäfer et al. 2013). In some cases, inoculation from a preculture in the late stationary phase was performed, without specifying a specific time (Lenz et al. 2018). Meanwhile, the use of glycerol stocks for inoculation of precultures is increasingly reported (Al-Shameri 2020; Sydow et al. 2017; Wilkening 2021). Similarly, there has been a move away from a fixed volume for inoculation (e.g. preculture to main culture of 1:20) (Jugder et al. 2016a) and more towards a fixed OD_436_ of 0.1 in the final volume (Kohlmann 2015; Wilkening 2021). To cover the aspect of a large volume of fit cells to inoculate larger main cultures two precultures are used (Wilkening 2021). This is also found in cultivations of other organisms, such as *Cupriavidus metallidurans* (Herzberg et al. 2015; Wiesemann et al. 2017). In 2013, Schiffels already pointed out that the condition of the precultures is considered to be critical for reproducibility (Schiffels 2013). Even though, each cultivation starts with a preculture and an optimal preculture management is a prerequisite for a sustainable process performance, preculture conditions have not been studied in detail so far and not every publication details their preparation. When applying either of these protocols, the use of fresh colonies of indeterminate size for inoculation of the preculture or growth of the preculture for a specific time, e.g. overnight or 48 h (Kohlmann 2015; Lu et al. 2016; Poladyan et al. 2019; Schäfer et al. 2016), without control of culture growth, resulted in different physiological states of the preculture cells, which consequently led to very poor reproducibility of the main cultures with different lag phases, protein yields and activities.

Thus, since there is a lack of concrete protocols for uniform precultures, we have taken it up to develop one, based on some of the existing methods. In this work, we established a uniform and reproducible protocol for precultures using two precultures, inoculation from defined cryostocks, a temperature of 30 °C, and switching from complex medium to minimal medium.

## Materials and methods

### Strain and media

*C. necator* HP80 (pGE771) (Lauterbach and Lenz 2013) was grown in nutrient broth medium (NB) containing 3 g meat extract l^−1^ and 5 g peptone from gelatin l^−1^, or fructose-nitrogen medium (FN) with 25 mM Na_2_HPO_4_ × 12 H_2_O, 11 mM KH_2_PO_4_, 0.8 mM MgSO_4_ × 7 H_2_O, 0.06 mM CaCl_2_ × 2 H_2_O, 0.01 mM FeCl_3_ × 6 H_2_O, 0.001 mM NiCl_2_ × 6 H_2_O, 37 mM NH_4_Cl and 4 g fructose l^−1^. Fructose-glycerol-nitrogen medium (FGN) is composed the same way as FN medium but contains a mixture of 2 g fructose and glycerol l^−1^ each.

### Culture conditions

Cultures were grown in 100 ml Erlenmeyer flasks in a final volume of 10 ml medium or 125 ml ultra yield flask with 25 ml medium under oxic conditions at 30 °C on an orbital shaker (Infors HT, Switzerland). 15 µg tetracycline ml^−1^ were used for selection. The optical density was measured in a spectrophotometer (Ultrospec 2100, Amersham Biosiences) at 436 nm (OD_436_).

### Generation of glycerol stocks

Glycerol stocks of *C. necator* HP80 were prepared as follows: Cells were grown in 100 ml Erlenmeyer flasks with 10 ml NB medium until the late exponential growth phase was reached. The culture was adjusted to OD436 of 2.8 with NB medium, mixed with equal parts of sterile 50% glycerol, aliquoted to 40 µl, flash-frozen and stored at -80 °C until further use. For inoculation of 10 ml NB preculture 40 µl of the cryostock was used.

## Results and discussion

### Growth from cryostocks works best in NB medium

The development of an efficient and robust bioprocess already starts with the preculture for the main process. A preculture must not only be reproducible, but also provide sufficient material of vital cells in the shortest possible time so that the main culture can grow quickly without long lag phases to ensure cost-effective production. Most critical in this regard are the adaptation times of the cells after transfer from a glycerol stock to the preculture and the transfer from the preculture to the main culture.

**Fig. 1:**
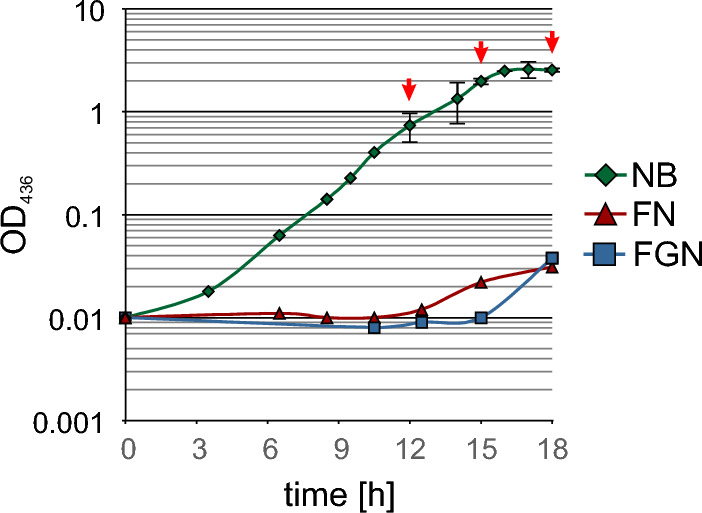
Growth of *C. necator* HP80 in different media inoculated from a NB cryostock. Precultures were inoculated with 40 µl cryostocks in 10 ml of each medium. Growth curves recorded by measurement of OD_436_ were performed in NB medium (green rhombuses), FN medium (red triangles) and FGN medium (blue squares). The arrows indicate time points for inoculation of the 2nd preculture

We tested three different media for their suitability as preculture. On the one hand, FN and FGN mineral salt medium, which were supposed to be used for the main cultures, and on the other hand NB, which had already been used to produce the cryostock. To ensure reproducible cultures, all cultivations were started from standardized cryostocks (see Material and Methods). Precultures in 10 ml NB, FN or FGN medium were inoculated with 40 µL each from the cryostocks in 100 ml shake flasks and cultured with vigorous shaking for 18 h at 30 °C. Samples were taken every 1 to 2 h to follow growth indicated as increase in optical density (OD_436_) (Fig. 1). Both the FN and FGN mineral salt media cultures showed almost no growth after 18 h and reached final ODs of only 0.03 and 0.04, respectively. This might be attributed to the slow adaptation of the cultures to these media, which is indicated by extremely long lag phases of 12 and 15 h, respectively (Fig. 1). In contrast, in NB medium, exponential growth started after about 3–4 h and stationary phase was reached after about 16 h (Fig. 1). The complex additives in NB medium allow a faster adaptation of the cells from the cryostock to the conditions in the liquid culture. Thus, the NB culture was chosen for inoculation of a second preculture instead of prolonged cultivation in FN or FGN.

### Inoculation of the second precultures should be done from 12–15 h first precultures.

**Fig. 2:**
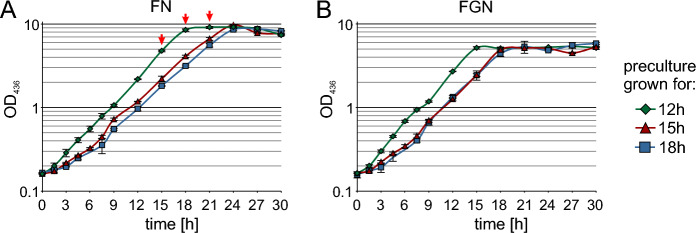
Growth of *C. necator* HP80 in FN (**a**) and FGN medium (**b**) inoculated from a 1st NB preculture. The cells were obtained during exponential growth phase (green rhombuses), late exponential phase (red triangles) or stationary phase (blue squares). Cell growth was recorded over 30 h of cultivation at 30 °C by shaking at 250 rpm. All cultivations were done in triplicates. The arrows indicate time points for inoculation of the main culture

Three time points were chosen to inoculate a second precultures in either FN or FGN mineral salt medium from the NB preculture. These time points correspond to the exponential growth phase after 12 h at an expected OD_436_ of approximately 1, the late exponential phase after 15 h at an expected OD_436_ of 2, and the early stationary phase after 18 h with an expected OD of 2.5. Samples were taken at different time points and OD_436_ was measured to follow growth (Fig. 2).

All cultures show comparable growth and reach similar OD_436_ maxima of approx. 9 in FN medium and 5 in FGN medium, respectively, irrespective of the duration of the 1st preculture. However, the extension of the 1st preculture leads to a slower growth of the 2nd preculture. Cultures inoculated from a 12-h preculture reach stationary phase after 15 and 18 h in FGN and FN medium, respectively, while cultures inoculated from 15- or 18-h precultures require 3 and 6 h longer to reach stationary phase in FGN and FN medium, respectively (Fig. 2). The slower growth can be attributed to a reduced fitness of the cells that leads to a prolonged lag phase when older precultures are used. Consequently, a 2nd preculture in FN medium inoculated from a 12 h first preculture in NB medium was found to be optimal.

### Main cultures should be inoculated from 14–17 h second precultures.

**Fig. 3:**
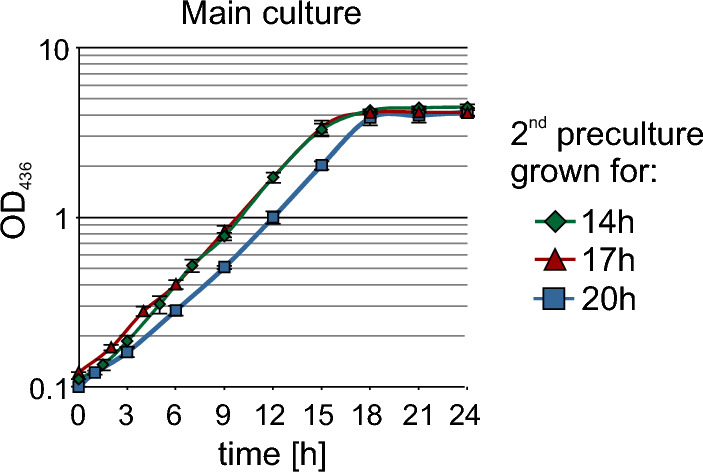
Main cultures of *C. necator* were grown in 25 ml FGN medium in 125 ml baffled shake flasks. They were inoculated to an OD_436_ of 0.1 after 14 h (green rhombuses), 17 h (red triangles) and 20 h (blue squares) of growth of the second preculture in FN medium. Growth was detected by measurements of OD_436_. All cultivations were done in triplicates

The first and second precultures were prepared as described above. After 14 h, 17 h and 20 h, a main culture of 25 ml was inoculated from the second preculture in a 125 ml ultra yield flask and the growth curve was recorded by OD_436_ measurement in the spectrophotometer (Fig. 3).

The two cultures inoculated from 14 and 17 h precultures grew nearly identical and slightly faster than that inoculated from the 20 h preculture with growth-rates µ of 0.23 h^−1^, 0.23 h^−1^ and 0.22 h^−1^. Both cultures reached stationary phase at an OD of about 4 after 15 h of cultivation. At that time the culture started from the 20 h preculture reached only about half of the OD_436_ from the other two cultures and reached stationary phase with similar OD_436_ of 4 only after 18 h. For inoculation of the main culture it was set that a second preculture should be cultivated between 14 and 17 h to gain fast and high growth of the main culture.

Consequently, a 12 h first preculture in NB medium followed by a 14 h second preculture in FN medium where found to be optimal for fast and reproducible growth of the main culture. The inoculation of the main culture with exponentially growing cells is more favorable, as adaptation to the fresh medium occurs more easily and faster. In contrast inoculation with cells from the late stationary phase tends to have negative effects on the subsequent growth of the main culture, characterized by a prolonged lag phase. Compared to single preculture systems in which the cells are cultivated for 48 h (Crépin et al. 2016; Wilkening 2021), or until the later stationary phase is reached (Lenz et al. 2018), the two-preculture system allows faster growth, due to the decoupling of the two critical adaptation steps, i.e. the transition from cryostock to growing liquid culture and the transition from complex medium to mineral salt medium.

## Conclusion

**Fig. 4:**
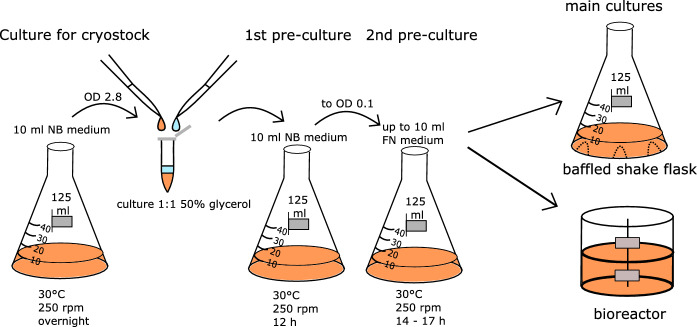
Protocol of optimized preculture procedure. A cryostock is prepared by cultivation of *C. necator* in 10 ml NB medium to an OD_436_ of 2.8 and mixing of the culture 1:1 with 50% sterile glycerol. A first preculture is inoculated with 40 µl cryostock in 10% filled shakeflask with NB medium and grown for 12 h at 30 °C and 250 rpm shaking. Then a second preculture is inoculated to OD_436_ 0.1 from the first preculture at 10% filling of the shakeflask and cultivated for 14 h to 17 h at 30 °C and shaking with 250 rpm

The present work addresses an important aspect of bioprocess development, the preculture management, which is often overlooked or underestimated, but is nevertheless important for successful and economic process design. Based on our results, we propose a two-stage preculture to be optimal (Fig. 4).

The major difficulty of single-stage precultures is that the critical transitions in the physiology of the cells, which occur when changing from cryostock or agar plate to liquid culture and from complex medium to the mineral salt medium used in the main culture, have to occur simultaneously. Both changes require an adaptation of the cell physiology and are accompanied by a more or less long growth arrest, which is represented by a prolonged lag phase. This problem is no longer apparent when using the two preculture scheme. The decoupling of the required two adaptions is advantageous as it allows faster adaptation of the cellular physiology thus reducing the overall preculture time to less than 30 h. Moreover, the possibility to use log-phase cells for inoculation of the main culture nearly abolishes a lag phase in the main culture thus further reducing the overall process duration which is beneficial for process economy. Furthermore, the almost negligible differences in growth of the individual cultures demonstrates the high reproducibility of our protocol, thus allowing accurate prediction of the time and culture volume required for inoculation of the main culture, allowing additional resource savings, e.g. reactor volume and culture medium.

In view of the increasing demand for alternative green energy, hydrogenases will be more and more in the focus of research. At the same time, the biotechnological production of hydrogenases must be improved in order to make these enzymes available in sufficient quantities for cost-effective application also on an industrial scale. The preculture management presented here could represent a first contribution to the development of such a process.

## Data Availability

The datasets generated during and/or analysed during the current study are available from the corresponding author on reasonable request.
